# Ultrasound-Assisted Extraction of Phenolic Compounds from Celtuce (*Lactuca sativa* var. *augustana*) Leaves Using Natural Deep Eutectic Solvents (NADES): Process Optimization and Extraction Mechanism Research

**DOI:** 10.3390/molecules29102385

**Published:** 2024-05-19

**Authors:** Shanshan Li, Guangyu Wang, Junjie Zhao, Penghui Ou, Qingping Yao, Wei Wang

**Affiliations:** 1 School of Perfume & Aroma and Cosmetics, Shanghai Institute of Technology, Shanghai 201418, China; liss@sit.edu.cn (S.L.); 216071132@mail.sit.edu.cn (G.W.); 216072136@mail.sit.edu.cn (J.Z.); 226072143@mail.sit.edu.cn (P.O.); 2 Institute of Mechanobiology & Medical Engineering, School of Life Sciences & Biotechnology, Shanghai Jiao Tong University, Shanghai 200240, China; qpyao@sjtu.edu.cn

**Keywords:** ultrasonic-assisted extraction, natural deep eutectic solvent, phenolic compounds, celtuce (*Lactuca sativa* var. *augustana*) leaves, molecular dynamics

## Abstract

Natural deep eutectic solvents (NADESs), as emerging green solvents, can efficiently extract natural products from natural resources. However, studies on the extraction of phenolic compounds from celtuce (*Lactuca sativa* var. *augustana*) leaves (CLs) by NADESs are still lacking. This study screened the NADES L-proline-lactic acid (Pr-LA), combined it with ultrasound-assisted extraction (UAE) to extract phenolic compounds from CLs, and conducted a comparative study on the extraction effect with traditional extraction solvents. Both SEM and FT-IR confirmed that Pr-LA can enhance the degree of fragmentation of cell structures and improve the extraction rate of phenolic compounds. Molecular dynamics simulation results show that Pr-LA can improve the solubility of phenolic compounds and has stronger hydrogen bonds and van der Waals interactions with phenolic compounds. Single-factor and Box–Behnken experiments optimized the process parameters for the extraction of phenolic compounds from CLs. The second-order kinetic model describes the extraction process of phenolic compounds from CLs under optimal process parameters and provides theoretical guidance for actual industrial production. This study not only provides an efficient and green method for extracting phenolic compounds from CLs but also clarifies the mechanism of improved extraction efficiency, which provides a basis for research on the NADES extraction mechanism.

## 1. Introduction

Celtuce (*Lactuca sativa* var. *augustana*), also called stem lettuce, Chinese lettuce or celtuce, is a cultivar of lettuce grown primarily for its thick stem and used as a vegetable and medicinal plant. It is especially popular in China, but it is not commonly consumed in European countries [1]. Celtuce is an abundant source of bioactive compounds, including phenolic compounds, glycosylated flavonoids, sesquiterpene lactones (e.g., lactucin and lactucopicrin), carotenoids, group B vitamins, ascorbic acid, and tocopherols [2]. Celtuce leaves (CLs), a readily available and untapped byproduct, are rich in phenolic compounds that display diverse biological properties that reduce the risk of cardiovascular disease, cancer, chronic inflammation, and aging, as well as improve immunity and protect vision [1]. However, the traditional extraction procedure for plant leaves with conventional solvents (methanol, ethanol, ethyl acetate, etc.) raises some concerns related to, for example lower extraction yields, lower contents of active constituents in the extracts, higher energy consumption, and lower environmental friendliness [3]. Therefore, it is urgent to develop highly efficient, green, and eco-friendly methods for the extraction of phenolic compounds from CLs.

Today, the efficient extraction of natural products from biomass using green and environmentally friendly solvents is considered an important area of concern in the food, pharmaceutical, and cosmetic industries. Natural deep eutectic solvents (NADESs) have garnered significant attention as potential green solvents, attracting interest in various industrial fields. NADESs are a generation of new and revolutionary green solvents, which share characteristics with ionic liquids (ILs). Indeed, previous studies have highlighted that ILs often exhibit poor biodegradability, and coupled with their potential toxicity, ILs may create environmental hazards, affecting ecosystems and living organisms. ILs are also associated with higher production costs, limiting their economic feasibility for certain applications [4,5]. In contrast to ILs, NADESs offer several advantages that make them well suited as extraction solvents, as follows: NADESs can be prepared using simple and cost-effective methods and exhibit high solubility for a wide range of compounds [6,7,8]. NADESs are also considered environmentally friendly due to their natural and biodegradable components, which is in line with the green concept of sustainable development [9,10]. The formation of NADESs usually involves of a hydrogen bond acceptor (HBA) and a hydrogen bond donor (HBD). The most commonly used HBAs currently are choline chloride, proline, and betaine, which are all considered ecofriendly and biocompatible molecules [11]. HBDs often include polyols, amines, carboxylic acids, and sugars [12]. The diversity of plants and the stochastic nature of NADESs contribute to the lack of versatility in using a specific NADES for the extraction of natural products from plants. Given this variability, customized NADESs are essential to optimize the extraction of phenolic compounds. By customizing the composition of a NADES, we can improve the extraction efficiency of phenolic compounds [13].

While using green-solvent NADESs to extract natural products, we also hope to combine them with green extraction technology to minimize the impact on the environment and reduce the use of harmful solvents or chemicals during the extraction process [14]. Traditional heating extraction (HE) often requires longer extraction times and higher extraction temperatures [15]. Compared with traditional extraction methods, green extraction technologies take less time, use fewer harmful organic solvents, and have higher extraction rates [14]. Common green extraction methods include ultrasound-assisted extraction (UAE), microwave-assisted extraction (MAE), supercritical fluid extraction (SFE), enzyme-assisted extraction (EAE), and pressurized extraction (PEFE) [14,16]. Among them, MAE generates excessive heat, which may lead to the thermal degradation and changes in the structure and properties of biologically active substances [17]. SFE, EAE and PEFE all have higher production or instrument maintenance costs, and they are not conducive to actual large-scale production [14]. Compared with the above methods, UAE not only reduces the use of solvents and shortens the extraction time but also destroys the structure of plant cell walls and improves the mass transfer efficiency between the solvent and cell matrix, thus improving the extraction rate of biologically active substances [18]. On the basis of the recognized advantages of NADESs and UAE, this study combined NADESs with UAE to extract phenolic compounds from CLs.

Therefore, this study screened out a green and efficient NADES solvent, L-proline lactic acid (Pr-LA), and combined it with UAE to extract phenolic compounds from CLs. SEM was used to observe the morphological changes of the sample powder before and after extraction, and FT-IR was used to analyze the functional groups of the extracted compounds. A molecular dynamics simulation was used to analyze the interactions between phenolic compounds and solvent molecules in Pr-LA, revealing the potential mechanism underlying the extraction of phenolic compounds by Pr-LA. Single-factor experiments and Box–Behnken design were used to optimize the process parameters of Pr-LA combined with UAE to extract TPC from CLs. Secondary kinetics research on the extraction process provides theoretical guidance for actual industrial production. In summary, this study aimed to establish an efficient and green method to extract phenolic compounds from CLs using NADES (Pr-LA) combined with UAE, and reveal the potential mechanism of action between Pr-LA and phenolic compounds.

## 2. Results and Discussion

### 2.1. Screening of NADES

**Figure 1:**
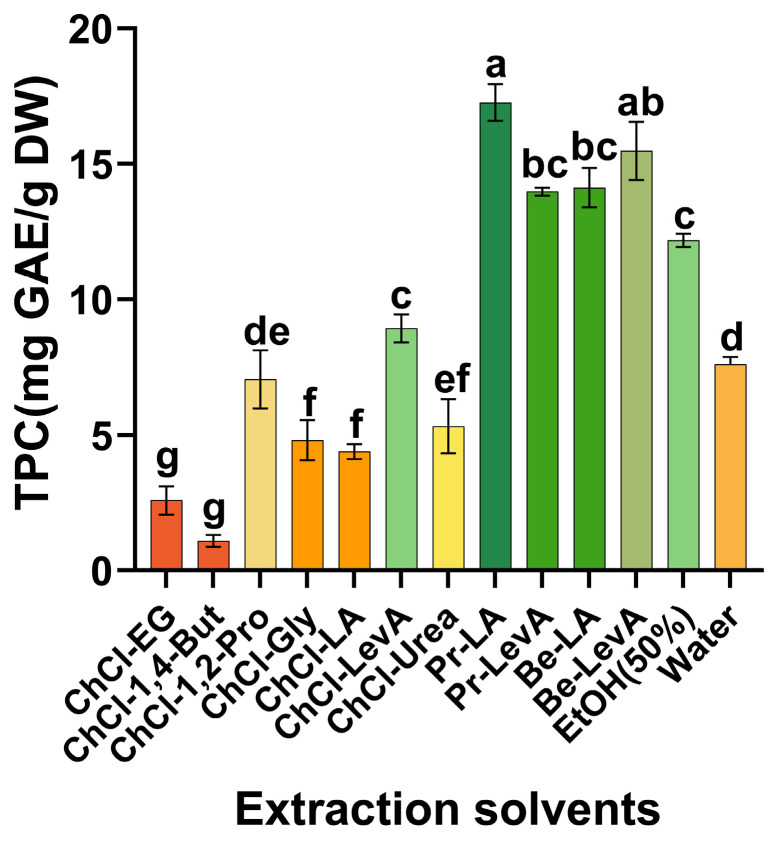
TPC in CLs extracted with different solvents. Different lowercase letters represent significant differences in the TPC extracted from CLs with different extraction solvents.

The components of NADESs are usually natural sources. Common HBAs include quaternary ammonium salts and amino acids. Common HBDs include polyols, organic acids, amides, and sugars. Two or more of these compounds combine with a certain proportion of water to form an eutectic liquid [19]. As a component of NADES, water not only reduces the viscosity of NADES and increases mass transfer efficiency of the solvent [20], but also enables NADES to form a stronger hydrogen bond network [21]. In terms of the selection of HBAs, we chose the common choline chloride, L-proline and betaine as the three major systems in this study [22]. Regarding the selection of HBDs, the extraction rate of phenolic compounds by sugar-based NADES is lower than that of polyol-based, organic acid-based and amide-based NADES [23]. Therefore, we selected polyols, organic acids and amides as HBDs for NADESs. The molar ratios of HBA:HBD of 1:1 and 1:2 are chosen by most studies [21]. However, in the betaine system, when the molar ratio of Be-LA to Be-LevA is 1:1, a liquid cannot be formed. NADESs can be formed as a uniform and transparent liquid when the molar ratio is 1:2 [24]. The same situation also occurs in the choline chloride system. When the molar ratio of ChCl-LevA is 1:2, the NADES can be formed as a uniform and transparent liquid [25]. In order to screen out the NADES with the highest efficiency in extracting phenolic compounds from CLs, we prepared 11 NADESs (Table 1). In addition to NADESs, 50% EtOH and water were employed as traditional solvents for comparative analysis. The TPC served as a pivotal metric for evaluating the extraction efficiency. Figure 1 illustrates the influence of various solvent types on the TPC. Among them, DES8, DES9, DES10, and DES11 have extraction rates for phenolic compounds from CLs exceeding 50% EtOH and water. The hydrogen bond alkalinity of an NADES is higher, which allows it to better penetrate plant cell walls and promote interaction between an NADES and plant cellulose chains, thereby improving the mass transfer efficiency of the solvent and increasing its extraction rate [21]. The extraction efficiencies of NADESs in the proline and betaine systems as solvents significantly surpass those of the choline chloride system. This may be because when an organic acid is introduced into the system, L-proline will be protonated as an HBA, thereby forming a positively charged quaternary ammonium group and counter-anion [26]. The quaternary ammonium group can solvate phenolic compounds in the hydrogen bond network, and the counter-anion and HBD provide an anhydrous solvation shell for phenolic compounds, which together improve the solubility of phenolic compounds [27]. At the same time, some studies have proven that organic acids can effectively dissolve lignin and cellulose in plant matrices, thereby significantly enhancing the extraction efficiencies of phenolic substances [28]. However, the Cl- in choline chloride may be wrapped by the hydroxyl and carboxyl groups in lactic acid, thus hindering the interaction between polyphenols and Cl^−^, thereby reducing the extraction yield of phenolic compounds [29]. Compared with the extraction effect of Be-LevA in the betaine system, Pr-LA shows a slight advantage. Different NADESs have different physical properties, such as viscosity, density, etc., which greatly affect the extraction of natural products. Viscosity is a key factor affecting the extraction effect [30]. High viscosity will reduce the mass transfer rate of the solvent and reduce the extraction efficiency of natural products [31]. Studies show that the viscosity of Pr-LA is much lower than that of Be-LevA, which may be the reason for the slight extraction advantage of Pr-LevA [32]. At the same time, the production cost and raw materials of levulinic acid are high, the production output is low, and recycling is difficult [32]. The high economic cost is not conducive to actual industrial production, so we did not choose Be-LevA as the extraction solvent. From a safety standpoint, lactic acid is deemed compatible with formulations in the food, pharmaceutical, and cosmetic industries [25]. On the basis of the extraction efficiency of phenolic compounds and the safety of the extraction solvent, we selected proline-lactic acid (Pr-LA) as the NADES solvent for extracting phenolic compounds from CLs.

### 2.2. Microstructural Analysis

**Figure 2:**
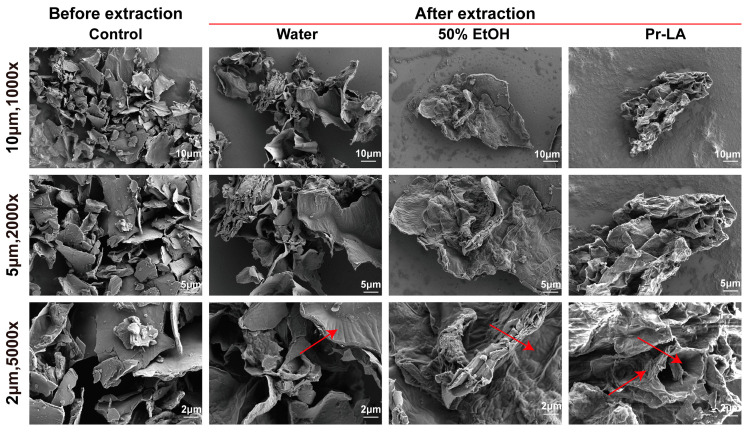
SEM analysis of residues before and after extraction of CLs using ultrasound-assisted combination with different solvents (The red arrow indicates the surface microstructural changes of the powder sample).

Solvents extract phenolic compounds from the cell matrix by destroying plant cells [33]. In order to explore the degree of damage to plant cells by different extraction solvents, scanning electron microscopy (SEM) was utilized to examine and explain the microstructure of CLs powder samples before and after extraction. The surface morphology of the sample before undergoing UAE exhibited a very smooth texture, with no obvious damage to the integrity of the cell walls. Its original structure was extremely well preserved (Figure 2). However, the microstructure of the CLs powders changed significantly after extraction, and the surface morphology of the samples incurred damage. In comparison to the untreated sample, the water-treated sample exhibited no significant changes in appearance. After extraction with 50% EtOH, the sample’s surface became rough with apparent wrinkles. Notably, the sample extracted with Pr-LA displayed more obvious pore and crack structures. This observation suggests that Pr-LA is more effective in disrupting the cell structure of the sample. Research indicates that direct contact between NADES solvents and intracellular compounds can enhance solvent penetration, leading to an improved extraction efficiency [34]. This study is consistent with the results reported by Wang et al., whereby NADESs can extract more phenolic compounds from partridge leaf tea by destroying the cell structure of the tea leaves [35].

### 2.3. FT-IR Analysis

**Figure 3:**
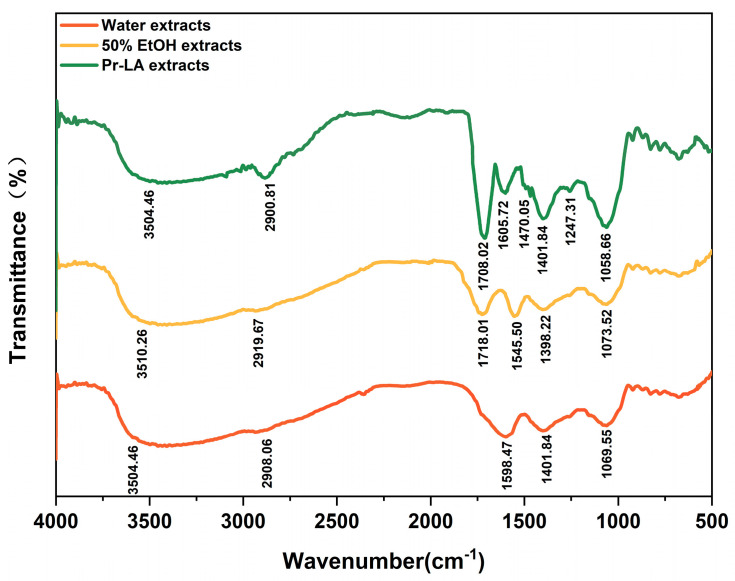
FT–IR spectra of corresponding extracts in three different solvents.

In order to observe the effect of Pr-LA solvent on the structure of the extract and compare it with the traditional extraction solvent of 50% EtOH and water, FT-IR scanning of extracts was carried out (Figure 3). It is evident that the water, 50% EtOH, and Pr-LA extracts exhibited expansive characteristic absorption peaks at around 3500 cm^−1^, which is attributed to the stretching vibration of -OH phenolic hydroxyl [36]. These three extracts displayed subtle variations at the wavenumbers 2900.81 cm^−1^, 2919.67 cm^−1^, and 2908.06 cm^−1^, which may be attributed to the aromatic C-H stretching vibration of the phenolic compounds. While in comparison, the Pr-LA extract showed a more obvious peak shape [37]. The absorption peaks of the 50% EtOH extracts and the Pr-LA extracts were observed at around 1700 cm^−1^. Notably, the peaks of the Pr-LA extracts at 1708.02 cm^−1^ displayed more pronounced fluctuations. These nearby fluctuations were attributed to the stretching vibration of the C=O carbonyl group, suggesting higher contents of phenolic substances in the Pr-LA extracts [38]. The absorption peak fluctuations at 1598.47 cm^−1^, 1545.50 cm^−1^, 1605.72 cm^−1^, and 1470.05 cm^−1^ were caused by the presence of an aromatic-ring conjugated structure or the C=C double bond [39]. The water, 50% EtOH, and Pr-LA extracts had strong specified peaks at 1401.84 cm^−1^, 1398.22 cm^−1^, and 1401.84 cm^−1^, which may be due to the presence of benzo-γ-pyrone structures in the flavonoids, flavones, and isoflavones [40]. The observed fluctuations in the range of 1250 to 1050 cm^−1^ in the infrared spectrum describe the presence of C-O stretching vibrations [39]. The characteristic peak fluctuations observed in the FT-IR spectra of the three extracts exhibited slight shifts. However, the overall shapes and positions remained essentially similar. This consistency suggests that the three extracts share similar compositions and all contain phenolic compounds. Some bands observed in the Pr-LA extracts exhibited higher intensities, additional peaks, and peak shifts, which indicates that Pr-LA improves the extraction effect of phenolic compounds to a certain extent.

### 2.4. Mechanism Analysis of Pr-LA Extracting Phenolic Compounds from CLs

#### 2.4.1. LC-MS Screening of Small Molecule Compounds in CLs

In order to better explore the mechanism of Pr-LA’s extraction of phenolic compounds, we used LC-MS to identify the phenolic compounds in CLs. Li et al. reported that different extraction methods will lead to different types of extracted phenolic compounds [22]. However, using the same extraction method, NADESs do not destroy the structure of the compounds but only increase the extracted content of the compounds [41]. Therefore, we selected the Pr-LA extract for the LC-MS identification analysis.

The mass spectrometer operates in two distinctive modes: positive ion and negative ion. Because of the abundant presence of phenolic compounds in CLs, a tendency for proton loss was observed in the negative ion mode. Consequently, the experimental protocol was executed in the negative ion mode to capture and analyze ion fragments of phenolic compounds. 

As illustrated in Figure S1 and Table S1, our analysis successfully identified 10 distinct phenolic compounds. Compound 1, as depicted in the chromatogram, exhibited a deprotonated ion [M–H]^−^ with an *m*/*z* of 169.0131. Compound 1 was identified as gallic acid by comparison with established standards. Peak 2 revealed a parent ion with an *m*/*z* of 353.0867. The software conducted a structural analysis, suggesting a possible formula of C_16_H_18_O_9_. Collision-induced dissociation in the secondary mass spectrum produced an *m*/*z* of 191.0553, probably due to the molecular ion losing its C_9_H_7_O_3_ structure of *m*/*z* 162.0233. This clear pattern led to the identification of the compound as chlorogenic acid. In peak 3, the primary mass spectrometry scanning detected [M–H]^−^ parent ions with an *m*/*z* of 289.0706. Based on the highest-scoring formula, the software assigned C_15_H_14_O_6_, the same formula as catechin. The subsequent secondary mass spectrometry revealed the presence of an *m*/*z* of 245.0819 and an *m*/*z* of 109.0284 ion fragments, further confirming that these compounds were catechin [42,43]. In peak 4, a precursor ion with an *m*/*z* of 179.0339 was observed. Subsequent collision-induced dissociation in the secondary mass spectrum led to the loss of a -CO_2_ group, resulting in a fragment ion peak at *m*/*z* 135.0439. It can be inferred that the compound may be caffeic acid [44]. At the same time, compared with the standard product, it was confirmed that the compound was caffeic acid. The presence of rutin in the extract was identified by comparison with analytical standards. During the mass spectrometry analysis of component 6, a deprotonated ion [M–H]^−^ at *m*/*z* 462.0793 was observed, corresponding to the molecular formula C_21_H_19_O_12_. The literature shows that isoquercitrin will lose glucoside and form an aglycone ion fragment of *m*/*z* 301.0265. At the same time, *m*/*z* 300.0280 is speculated to be the aglycone ion losing one H to form a free radical aglycone ion fragment, and *m*/*z* 271.0252 may be the ion fragment obtained by removing COH from the free radical aglycone ion. The structural presentation of this fragment exhibited remarkable consistency with that of isoquercitrin. Consequently, component 6 was confidently identified as isoquercitrin [45,46]. In peak 7, a precursor ion with an *m*/*z* of 193.0495 was identified. The software analysis suggested a possible molecular formula of this substance is C_10_H_10_O_4_. Further examination in the secondary mass spectrum revealed an explicit dissociation pattern, as follows: the parent ion underwent collision-induced dissociation, causing the loss of one -CO_2_ group to generate a fragment ion at an *m*/*z* of 149.0596. Additionally, the loss of one -CH_3_ group yielded a fragment ion with an *m*/*z* of 178.0262. This fragmentation pattern is consistent with the cleavage process observed in the ferulic acid literature [44,47], thus identifying the compound as ferulic acid. By comparing the observed fragment ions with the primary mass spectrometry information and secondary mass spectrometry information in the standard, peak 8 was preliminarily identified as luteolin. In peak 9, the [M–H]^−^ parent ion was identified with an *m*/*z* of 271.0618. After a database comparison, it was initially speculated that the compound was naringenin. Further verification with secondary mass spectrometry revealed fragmentation of the precursor ion, producing fragments with *m*/*z* values of 119.0489 and 151.0024. This fragmentation pattern is fully consistent with the documented processes reported in the naringenin literature [48,49]. The precursor ion peak of compound 10 was observed with an *m*/*z* of 315.0499, and secondary fragmentation produced characteristic ions of *m*/*z* 193.0458 and *m*/*z* 165.0369. The fragmentation pattern correlates well with the rhamnetin process reported in the literature. Therefore, compound 10 was identified as rhamnetin [50]. The LC-MS results show that 10 phenolic compounds were identified in the CLs extract.

#### 2.4.2. Molecular Dynamics Simulation Analysis

In order to reveal the mechanism by which Pr-LA extracts phenolic compounds from CLs and to elucidate the reason for NADESs’ superior extraction efficiency over traditional solvents, we performed molecular dynamics simulations. Previous studies showed that the interaction between solvent molecules and solute molecules affects the solubility of solute molecules in the solvent, thereby affecting the extraction yield of solute molecules [51]. In order to research the dissolution behavior of phenolic compounds in solvents from an atomic perspective, we randomly selected a typical phenolic compound caffeic acid from 10 phenolic compounds, as a small molecule compound.

**Figure 4:**
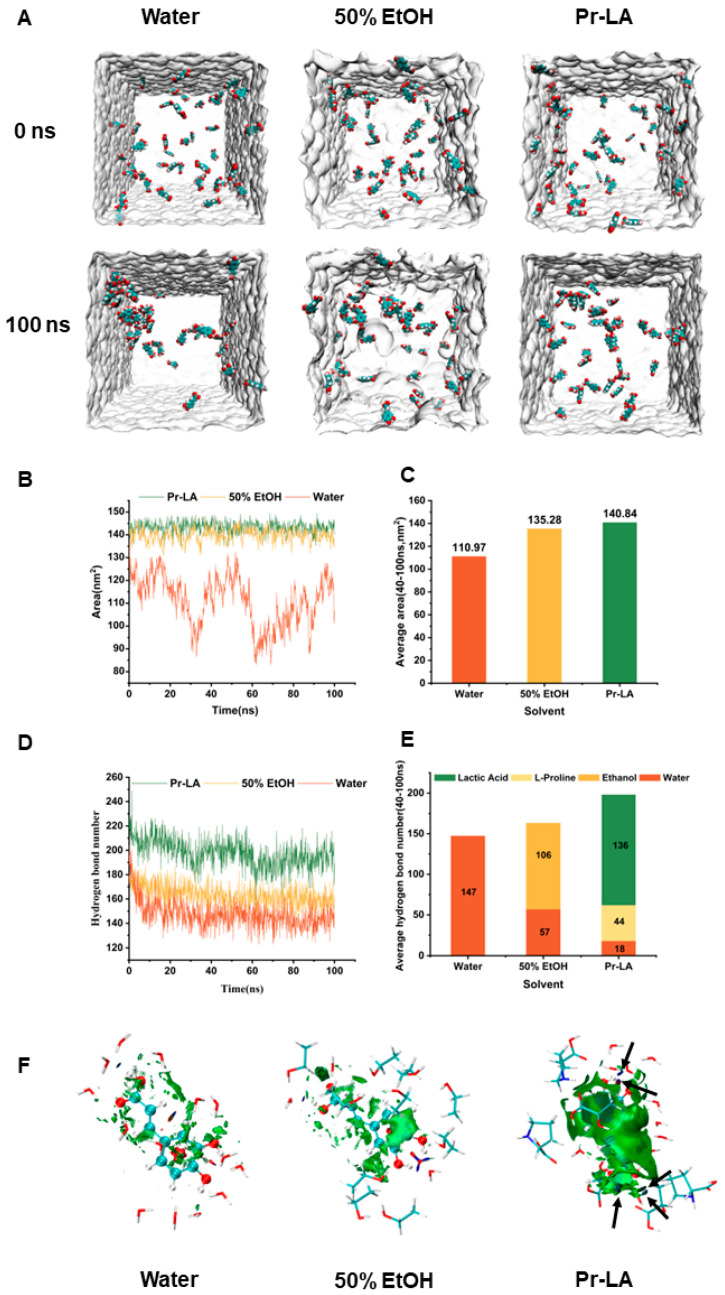
Study on the interaction of caffeic acid molecules with different solvents. (**A**) The intuitive state of caffeic acid molecules dissolved in different solvent systems at 0 and 100 ns, respectively, (**B**) Changes in the SASA of caffeic acid molecules in different solvents from 0 ns to 100 ns. (**C**) The average SASA of caffeic acid in different solvents from 20 ns to 100 ns. (**D**) The number of hydrogen bonds formed by caffeic acid molecules in different solvents changes from 0 ns to 100 ns. (**E**) The average number of hydrogen bonds formed by caffeic acid molecules in different solvents from 20 ns to 100 ns. (**F**) Average noncovalent interactions (aNCI) analysis (The black arrow represents the blue area of caffeic acid molecules in Pr-LA).

In this study, three different solvent systems, water, 50% EtOH, and Pr-LA, were selected for dissolution behavior analysis. Figure 4A is a visual diagram of the dissolution state of caffeic acid molecules in three different solvents at two time points of 0 ns and 100 ns. In water, caffeic acid molecules exhibit an aggregated state, indicating that caffeic acid molecules have a tendency to aggregate in water. In 50% EtOH, only a portion of the caffeic acid molecules assemble to form clusters. Notably, the distribution of caffeic acid molecules appears more disordered and dispersed in Pr-LA. This phenomenon indicates that caffeic acid molecules exhibit higher solubility in 50% EtOH and Pr-LA compared to water. Solvent accessible surface area (SASA) is a measure of the effective contact area between solute and solvent molecules in a solution. By analyzing the SASA of caffeic acid molecules in the three solvent systems, the degrees of contact between caffeic acid molecules and water, 50% EtOH, and Pr-LA can be quantitatively compared. As shown in Figure 4B, the dissolution curve of caffeic acid molecules in water showed obvious fluctuations, indicating that the solubility of caffeic acid molecules in water was poor. In comparison, the SASA values in 50% EtOH and Pr-LA showed a smoother curve. After 20 ns, the caffeic acid molecules in 50% EtOH and Pr-LA reached a stable dissolved state. The average SASA of caffeic acid molecules in the three different solvents within 20 ns–100 ns is shown in Figure 4C. Among them, the SASA of Pr-LA (140.84 nm^2^) was slightly higher than that of the SASA in 50% EtOH (135.28 nm^2^), and it significantly exceeded that of the SASA in water (110.97 nm^2^). This observed trend is consistent with the intuitive state diagram of the dissolution of caffeic acid molecules in the three systems. In Figure 4D, the dynamic evolution of hydrogen bond formation by caffeic acid molecules with the solvent is illustrated over the 0 ns to 100 ns period. The tendency of caffeic acid molecules to form hydrogen bonds in the three solvents becomes stable after 20 ns. Figure 4E shows the average hydrogen bonds formed between caffeic acid molecules and three different solvent molecules between 20 ns and 100 ns. Caffeic acid molecules form 147 hydrogen bonds in water, 163 hydrogen bonds in 50% EtOH, and 198 hydrogen bonds in Pr-LA. Caffeic acid molecules formed significantly more hydrogen bonds with Pr-LA compared to 50% EtOH and water, indicating the existence of a strong hydrogen bonding network between NADES and phenolic compounds. This strong network promotes interactions between solute and solvent molecules to increase solubility [51].

Figure 4F shows the average noncovalent interactions (aNCI) between caffeic acid molecules and three different solvent molecules. In the aNCI analysis, the dark blue area represents the extremely attractive hydrogen bond interaction, the blue–green area represents the π–hydrogen bond formed by the aromatic ring π electron area of the caffeic acid and the solvent molecules, and the green area represents the strong van der Waals force. The red area represents the steric hindrance effect. The caffeic acid molecule has the largest blue area in the Pr-LA, indicating that the hydrogen bond interaction formed between phenolic compounds and Pr-LA is the strongest. Studies showed that the stronger the hydrogen bond interaction between phenolic compounds and solvent molecules, the higher the solubility of phenolic compounds [52]. At the same time, the green area of caffeic acid molecules in Pr-LA is much larger than the green area of the caffeic acid molecules in 50% EtOH and water, indicating that the van der Waals interaction formed between caffeic acid molecules and Pr-LA is the strongest. Strong van der Waals interactions make solute molecules in NADESs more stable. This enhanced stability helps to increase the solubility of solute molecules in the solvent [53].

In summary, caffeic acid molecules exhibit higher SASA and more hydrogen bonds in Pr-LA, indicating that caffeic acid molecules have higher solubility in NADES. At the same time, the aNCI molecule reveals the interaction mechanism between caffeic acid molecules and Pr-LA solvent molecules. Specifically, caffeic acid exhibits stronger hydrogen bonds and van der Waals interactions in Pr-LA, which is the reason why the extraction efficiency of Pr-LA is better than traditional solvents. Therefore, Pr-LA is a green solvent with greater extraction potential than traditional solvents.

### 2.5. Optimization of the Extraction Process of TPC from CLs

#### 2.5.1. Single-Factor Experiment for Extraction of TPC from CLs

**Figure 5:**
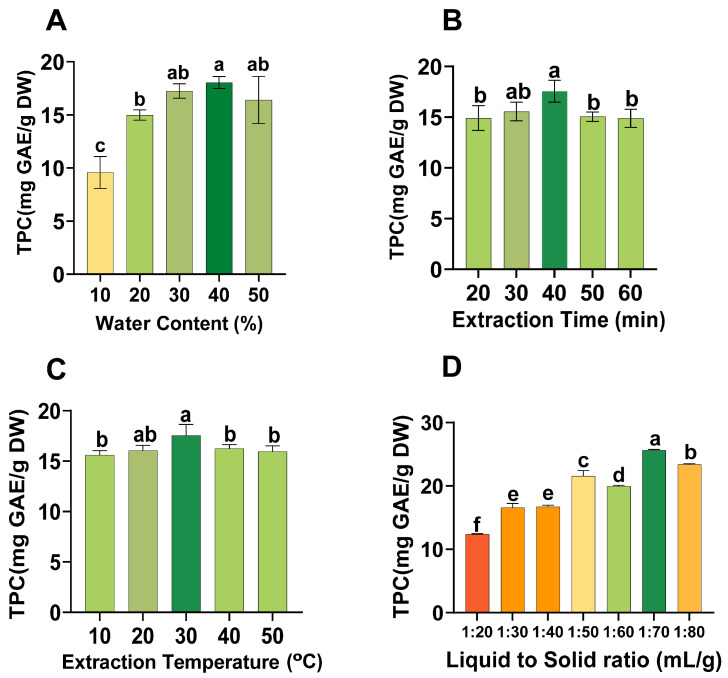
Single-factor experiment for extraction of TPC from CLs: (**A**) water content of NADES (%); (**B**) extraction time (min); (**C**) extraction temperature (°C); (**D**) liquid to solid. Different lowercase letters represent significant differences in TPC extracted from CLs under different experimental conditions.

To initially evaluate effects of different factors on TPC in CLs extract, a single-factor experiment was designed. The remaining experimental parameters were kept unchanged to specifically examine the influence of water content of NADESs on TPC in extract. The experimental outcomes are depicted in the Figure 5A. A gradual increase in TPC was observed with a gradual increase in the water content of the NADES. The NADES is a relatively viscous extraction solvent. The addition of water greatly reduces the viscosity of the solvent, thereby enhancing mass transfer effect [54]. Previous studies also provided evidence to support the view that the addition of water can effectively diminish the viscosity of NADES, thereby increasing the solubility of phenolic compounds and improving the extraction yield [55]. The TPC reached its peak when the water content of NADES reached 40%. Thereafter, the extraction yield of TPC by NADES showed a declining trend. Water is considered an effective polar solvent. However, excessive addition of water can weaken the hydrogen bonds in NADES and increase the polarity of NADES excessively, which may be the reason for decrease in the extraction efficiency of NADES in extracting TPC of CLs [56,57]. Therefore, 40% water content of NADES was selected for subsequent experiments.

Figure 5B illustrates the impact of extraction time on the yield of TPC. The extraction yield of TPC increased within the range of 10 to 40 min. This phenomenon can be attributed to the gradual diffusion of phenolic compounds within the plant cell matrix into the solvent over time [58]. At 40 min, the extraction yield of TPC reached the highest level. Following this peak, the extraction yield of TPC began to stabilize and exhibited a slight decrease. This is because when the mass transfer of NADES and phenolic compounds had essentially reached equilibrium, the phenolic compounds will be exposed to the air for a long time, which will cause a certain degree of degradation, and ultimately lead to a decrease in the extraction yield of TPC [59,60]. The experimental results show that the optimal extraction time for TPC from CLs is 40 min.

The extraction temperature is another key factor that significantly affects TPC extraction. Its influence on TPC is depicted in Figure 5C. As the extraction temperature increased from 10 °C to 30 °C, the TPC extraction yield increased continuously. Increased temperatures reduce the strength and stability of plant cell walls, making the phenolic compounds in plant cells more exposed to NADES [61]. At the same time, the increase in temperature will reduce the viscosity of NADES and then increase the mass transfer rate between solute and solvent molecules, which improves its extraction efficiency [62]. The extraction yield of TPC reaches its peak when the temperature reaches 30 °C. However, as the temperature increased to 50 °C, the extraction yield of TPC began to decrease. The occurrence of this phenomenon can be attributed to the fact that high temperatures trigger the degradation of phenolic compounds [63]. So the optimal extraction temperature is 30 °C.

In Figure 5D, it is obvious that the TPC gradually increased as the L/S ratio increases. Because as the L/S ratio increased, the solubility of the phenolic compounds in the NADES increased [64]. When the L/S ratio reached 70 mL/g, the extraction yield of the TPC demonstrated a tendency to stabilize. Once the solubility of a target compound reaches saturation, further increases in solvent volume cannot increase the solubility of the target compound. The TPC even showed a slight downward trend when the amount of solvent became excessive [39].

#### 2.5.2. Optimize Experimental Parameters Using RSM

In order to explore the interaction between important factors affecting TPC extraction and find the best conditions for process optimization, we conducted a Box–Behnken design (BBD) experiment. From the single factor experiment, it can be seen that the water content of NADES, extraction time, extraction temperature, and L/S ratio all have significant impacts on the extraction yield of the TPC. Therefore, these four influencing factors were determined as independent variables in the BBD experiment. The experimental design is shown in Table S2. After performing multiple regression analysis on the experimental data, the model is represented by a second-order polynomial equation. The polynomial equation with TPC as the response value is:

(1) $$\begin{array}{l} Y=24.46-1.33A+0.5559B+0.5826C+1.59D-1.19AB+0.8880AC\\ +0.8453AD-1.72BC-0.4831BD+0.0615CD-3.50A^{2}-1.87B^{2}-0.6470C^{2}-1.56D^{2} \end{array}$$

where *Y* is extraction yield of TPC (mg GAE/g DW), *A* is the water content of the NADES (%), *B* is the extraction time (min), *C* is the extraction temperature (°C), and *D* is the L/S ratio (mL/g).

As shown in Table 2, the *p*-value of the “Model” item is <0.0001 (*p* < 0.0001), indicating that the independent variables in the model had a significant impact on the response variable as a whole, which means that the model was successfully established; the *p*-value of the “Lack of Fit” item is not significant, signifying that the model had a good degree of fit, which adapts well to the observation data, and there was no obvious lack of fitting; the coefficient of determination (R^2^) of the model was 0.9602, demonstrating that the model can fit the observation data well, and the model had strong predictive ability. The difference between the adjusted R^2^ and the predicted R^2^ was less than 0.2, which indicates that the difference in the model’s performance between the training data and the test data was relatively small. It also means that the predictive capability of the model remains stable across different datasets and is not prone to significant fluctuations due to data changes. Simultaneously, the results from Table 2 reveal that the individual factors A and D exert a highly significant influence on the response value (*p* < 0.0001), while factors B and C exhibit a significant impact (*p* < 0.05). Among the model interaction terms, AB, AC, AD, and BC demonstrate a significant effect (*p* < 0.05), and BD and CD do not exhibit significant effects (*p* > 0.05). Additionally, all the quadratic terms, A^2^, B^2^, C^2^ and D^2^, exert a significant effect (*p* < 0.05).

In order to express the interaction between factors affecting TPC extraction more intuitively, we generated a three-dimensional response surface plot based on the regression model equation. When creating these plots, we ensured that two of the influencing factors were maintained at the middle level while observing the interaction of the remaining two influencing factors on TPC extraction. In the three-dimensional response surface diagram, the greater the curvature of the surface, the more obvious the interaction between the two influencing factors.

Figure S2A–C exhibit a consistent increasing trend in TPC extraction yield with increasing water content of the NADES. The inherently high viscosity of the NADES may prevent the efficient interaction between the solvent and the sample. The viscosity of the solvent can be weakened by introducing water into the NADES, thereby achieving the purpose of improving the extraction yield of the target product. Nonetheless, excessive addition of water hinders the interaction between the sample and solvent. Figure S2A,D,E collectively illustrate a clear increasing trend in the TPC extraction yield as the extraction time increased. The extension of the extraction time had a positive impact on the extraction yield of the TPC. However, when the maximum value was surpassed, the effect of the extraction time turned from positive to negative. The main reason behind this shift is that long extraction times lead to extended exposure times of the target product, resulting in a slight decrease in the yield. Figure S2B,D,F show that TPC extraction yield increases with increasing temperature. The increase in temperature will increase the solubility of the phenolic compounds and accelerate the mass transfer rate, thus increasing the extraction yield of TPC. Likewise, excessively high temperatures can lead to the degradation of active substances, resulting in reducing the extraction yield of TPC. Figure S2C,E,F show that the TPC extraction yield had a clear increasing trend with an increasing L/S ratio. The L/S ratio plays an important role in the conduction of active ingredients. Increasing the amount of solvent reduces the concentration of ingredients around the sample, favoring the diffusion of active compounds inside the cells into the solvent. However, adding too much solvent not only causes waste but also increases nontarget components. Therefore, when the L/S ratio reaches the maximum value, the TPC content will decrease slightly as the L/S ratio increases.

On the basis of the regression model’s optimization, the optimal extraction conditions for TPC are as follows: NADES water content of 41%, extraction time of 40 min, extraction temperature of 36 °C, and L/S ratio of 76 mL/g. The predicted yield under these conditions is 24.93 mg GAE/g DW. To validate the predicted yield results, three parallel experiments were conducted under the optimal extraction conditions, and the experimental yield was 25.17 mg GAE/g DW TPC. The experimental and predicted values of 24.93 mg GAE/g DW were very similar, and the difference between the predicted and experimental yields was only 0.95% (less than 5%). This shows that the model can, indeed, be used to extract TPC from CLs.

### 2.6. Extraction Kinetic Study

**Figure 6:**
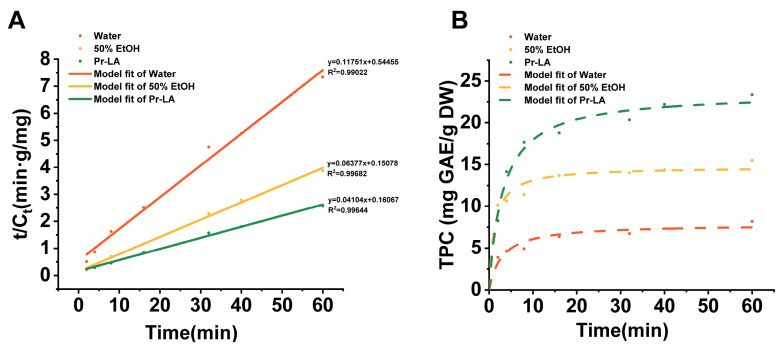
Second-order kinetic model (**A**) and extraction dynamic curve (**B**) of ultrasound-assisted extraction of TPC from CLs combined with P-LA.

In order to verify the extraction advantages of NADES in the actual production process, a kinetic study was conducted on the extraction process of TPC from CLs with water, 50% EtOH and Pr-LA under the extraction conditions of 2.5.2. Apply Equation (5) to process the experimental data and draw graphs. It can be seen from Figure 6A that the second-level kinetic model has a good fit for the process of extracting TPC from CLs, indicating that the second-level kinetic model can be used to describe the extraction process of TPC. The second-order kinetic model parameters Cs, h, and k (Table S3) were calculated based on the slope and intercept in the fitting equation in Figure 6A. “Cs” represents the saturation concentration of TPC (mg GAE/g DW). The larger the Cs value, the higher the solubility of phenolic compounds in the extraction solvent. “k” represents the extraction rate constant (g·min^−1^·mg^−1^), and a larger rate constant means reaching extraction equilibrium in a shorter time. From Table S3, it can be seen that during the extraction of TPC from CLs, the Cs value: Pr-LA > 50% EtOH > water, which means that the solubility of phenolic compounds in Pr-LA is higher compared with water and 50% EtOH. Previous studies have pointed out that the hydrogen bond network formed by hydrogen bond donors and hydrogen bond acceptors in NADES may be the reason for increasing the solubility of phenolic compounds [65]. However, the rate constant value “k” exhibits the order 50% EtOH > Water > Pr-LA. The solvent Pr-LA has a higher viscosity compared to 50% EtOH and water. High viscosity may reduce the mass transfer rate of phenolic compounds within the Pr-LA solvent system, which may be responsible for the difference in rate constants [66]. In Figure 6B, the inflection point of the fitting curve reflects the rate constant k. The earlier the inflection point occurs, the larger the rate constant k. This is consistent with the order of k values in Table S3. After the inflection point, the curve will become flat, which means that the TPC value reaches the saturation value, which also confirms the order of the Cs value. 

The extraction kinetics study not only proves that Pr-LA can improve the extraction yield of TPC in actual industrial production, but also provides theoretical guidance for actual industrial production.

## 3. Materials and Methods

### 3.1. Materials and Chemicals

Celtuce leaves (CLs) were procured from a local farmers market situated in Shanghai in December. CLs were carefully washed by hand and stored in a refrigerator at −18 °C for 2 days, then subjected them to freeze-drying process in a freeze dryer (TF-FD-1, Shanghai Zhefen Machinery Co., Ltd., Shanghai, China). Subsequently, the dried leaves were crushed into fine powder using an 800 Y mill (Wuyi Haina Co., Ltd., Jinhua City, China), and sifted through a 50-mesh sieve. The resulting dry sample powder was carefully stored at 4 °C for future use.

All analytical standards were provided by Shanghai yuanye Bio-Technology Co., Ltd. (Shanghai, China). Acetonitrile and formic acid were provided by Macklin Company (Shanghai, China). Choline chloride, L-proline, lactic acid and other chemical reagents were purchased from Alladdin (Shanghai, China).

### 3.2. Preparing and Screening of DESs

According to Table 1, Prepare 11 different NADES using the heating and stirring methods described above, mix HBA and HBD, then stir and heat at 80 °C, and add 30% water until a transparent and uniform stable state [3,67].

**Figure 7:**
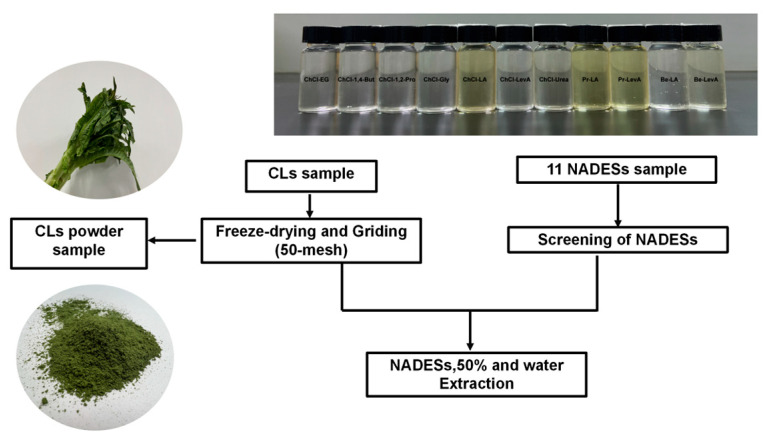
Diagram of sample processing steps before extracting phenolic compounds from CLs.

For preliminary evaluation of NADES, 0.5 g of CLs dry powder was added to 12.5 mL of NADES prepared above. Subsequently, the extraction was performed using a KQ-250DB ultrasonic instrument (Kunshan, Jiangsu, China) with an extraction time of 30 min, an extraction temperature of 30 °C, and a power of 200 W, followed by centrifugation at 10,000× *g* for 10 min. The resulting supernatant was carefully collected and subsequently diluted tenfold in preparation for subsequent analysis. Figure 7 shows the sample processing steps before extracting phenolic compounds from CLs.

### 3.3. Determination of Total Phenolic Content (TPC)

Total phenolic content (TPC) was determined following previously established methodologies with slight modification [68]. Briefly, 1 mL of the diluted extract was combined with 2.5 mL of a 10% (*v*/*v*) Folin-Ciocalteu solution, 7.5% (*w*/*v*) Na_2_CO_3_ solution was introduced, the mixture was then incubated in a dark environment at room temperature for 120 min. The resulting mixture was measured using a microplate reader at 760 nm and the results were expressed as gallic acid equivalents per gram of CLs dry weight (mg GAE/g DW). Gallic acid was used as the reference standard. The standard curve equation for gallic acid is Y = 0.009676 × X + 0.01791 (R^2^ = 0.9990).

### 3.4. Traditional Solvent Extraction of TPC from CLs

In this experiment, 0.5 g of CLs sample was mixed with 12.50 mL of conventional solvent (50% EtOH and water) in a 50 mL tube. Extraction conditions followed the experimental parameters outlined in Section 3.2. After centrifugation at 10,000× *g* for 10 min, the resulting supernatant was collected. Subsequently, a comparative analysis of TPC was performed on the extracted CLs extracts.

### 3.5. Scanning Electron Microscopy (SEM)

The extracted sample residues of three samples were washed alternately with ethanol and water until there was no ethanol smell. Then three extracted sample residues were dried. The experimental conditions were set to voltage of 3 KV, 60 µm objective aperture, and 6.8 mm working distance. The dried sample was firmly mounted on a silicon wafer, and prayed with gold using a sputtering coater for 45 s with a gold spraying temperature of 10 mA (Quorum SC7620, San Jose, CA, USA). The microstructure of the samples before and after extraction with different solvents (NADES, 50% EtOH, and water) were observed using a field emission scanning electron microscope (Hitachi, Tokyo, Japan). Samples were viewed at magnifications of 1000, 2000, and 5000.

### 3.6. Fourier Transforms Infrared Spectra (FT-IR)

The extract was freeze-dried for 48 h and ground into powder. KBr and the extract are mixed evenly and then pressed into thin sheets. The experimental parameters were set to scan 16 times. Subsequently, an FT-IR spectrometer (Thermo Fisher Scientific, Waltham, MA, USA) was used to scan in the wavenumber range of 4000–400 cm^−1^. The data were assessed using Omnic 9.0 software (OMNIC^TM^ Series, Thermo Scientific, USA) once all the spectra were collected.

### 3.7. Mechanism of Extraction of Phenolic Compounds from CLs

#### 3.7.1. Identification of Phenolic Compounds in Pr-LA Extracts by LC-MS

Qualitative analysis of phenolic compounds in Pr-LA extracts was performed using Ultimate 3000 UHPLC-Q Exactive (ThermoFisher Scientific, USA). An Eclipse Plus C18 chromatographic column (100 mm × 4.6 mm, 3.5 μm) was used for separation. The mobile phase contained acetonitrile (A) and a 0.1% aqueous solution of formic acid (*v*/*v*) (B). Gradient elution was used: 0–2 min, 95% B; 2–4 min, 80–95% B; 4–12 min, 80–85% B; 12–14 min, 50–85% B; 14–26 min, 0–50% B; 26–28 min, 0% B; 28–29 min, 0–95% B; 29–30 min, 95% B. Briefly, electrospray ionization (ESI) in negative ion mode was used, and the analysis included a first-level full scan and a second-level automatic scan. The ion source voltage was set to −4 kV, the capillary temperature was strictly controlled at 320 °C, and the working temperature of the auxiliary device was 300 °C. We used the software Xcalibur (Xcalibur^TM^ 2.0.7, Thermo Scientific) to analyze these data. For the accuracy of the results, we compared and analyzed the molecular ion peak and secondary fragmentation information simultaneously with analytical standards and data results from previous studies.

#### 3.7.2. Molecular Dynamic Simulation

Use the Pubchem website to collect and draw the original structures of molecules such as Caffeic Acid, L-proline, lactic acid, ethanol, etc. Use the OPLSAA force field online website to generate topological information for small molecules.

Boxes with dimensions of 10 nm × 10 nm × 10 nm containing caffeic acid molecules and different solvent molecules (proline-lactic acid and water, ethanol and water, water) were constructed using the Gromacs built-in inserter. Three solvent boxes containing caffeic acid were constructed: a solvent box using Pr-LA as solvents, and 1000 proline ions, 500 lactic acid molecules, 8309 water molecules, and 40 caffeic acid molecules were randomly added in proportion, the water molecules adopt the commonly used and accurate SPCE water model (the same below). For the solvent box with a solvent of 50% EtOH, 2000 ethanol molecules, 4755 water molecules, and 40 caffeic acid molecules were randomly added. In the solvent box using water as the solvent, a total of 16,581 water molecules and 40 caffeic acid molecules were randomly introduced.

Each simulation process was performed using the Oplsaa force field at 338.15 K and 1 atm, and the conjugate gradient method combined with the steepest descent method was used for energy minimization. Then, the system was initially equilibrated for 10 ns in constant pressure–constant temperature (NPT) simulation conditions. Finally, a final simulation process of 100 ns was performed under a constant NPT system.

This study used VMD 1.9.3 software (https://www.ks.uiuc.edu/Research/vmd/, accessed on 2 December 2022) and its own Tcl language to visualize and statistically analyze the simulation results. Statistical drawings were created using Origin 2021 software.

### 3.8. Design of Experiments

#### 3.8.1. Single-Factor Experiment

The experimental design was carried out according to previously established methods with certain modifications [69]. After selecting the NADES with the best extraction effect, a single-factor experiment was conducted to study the impact of each extraction factor on TPC. These single factors include the water content of NADES (10–50%), extraction time (20–60 min), extraction temperature (10–50 °C), and liquid-to-solid ratio (L/S ratio) (20–80 mL/g). In order to improve the accuracy of the experiments, all experiments were performed three times.

#### 3.8.2. RSM Experiments

Response surface methodology (RSM) is often used to optimize experimental parameters in extraction experiments [70,71]. The experimental design adopted a four-factor, three-level Box–Behnken design (BBD) with the goal of maximizing TPC. The four main influencing variables are water content of NADES, extraction time, extraction temperature and L/S ratio. Notably, all experiments were performed in triplicate. The response value TPC is expressed using a second-order polynomial equation, the equation of which is as follows:

(2) $$Y=\beta_{0}+\sum_{i=1}^{k}\beta_{i}X_{i}+\sum_{i=1}^{k}\beta_{\mathrm{ii}}X_{i}^{2}+\sum_{i=1}^{k}\sum_{j=i+1}^{k-1}\beta_{\mathrm{ij}}X_{i}X_{j}+\varepsilon$$

In the equation provided, “*Y*” signifies the response variable; “*X_i_*” and “*X_j_*” represent the independent variables; The coefficients *β*_0_, *β_i_*, *β_ii_*, and *β_ij_* correspond to the regression coefficients for the intercept, linear, quadratic, and interaction terms, respectively; *ε* is a random error.

### 3.9. Kinetic Model

The extraction process can be regarded as the reverse process of the adsorption process. Therefore, the basic principles of the adsorption kinetic equation are suitable for application in the field of extraction. Second-order kinetics have been effectively applied to elucidate the extraction kinetics of NADES [72,73,74]. The kinetic study of TPC extraction from CLs by NADES was conducted under RSM optimization conditions, which included a water content of the NADES of 41%, extraction time of 40 min, extraction temperature of 36 °C, and L/S ratio of 76 mL/g. Similarly, kinetic studies of TPC extraction were performed using water and 50% EtOH under the same UAE conditions as using NADES extraction. Extracts were collected at specific time intervals and subsequently analyzed for TPC yield. According to previous studies, the dissociation rate of TPC from CLs can be calculated by the following Equation (3):

(3) $$\frac{dCt}{dt}=k{\left(Cs-Ct\right)}^{2}$$

In the equation provided, “*C_t_*” represents the TPC yield (mg GAE/g DW) expressed in “t” (minutes), “*C_s_*” represents the saturation concentration of TPC in CLs (mg GAE/g DW), and “*k*” represents the extraction rate constant (g·min^−1^·mg^−1^). Integrating the rate law of this second-order model over time “t” from 0 to “t” and concentration “*C_t_*” from 0 to “*C_t_*” leads to the formulation of Equation (3). This equation can then be converted into a linearized representation, expressed as Equation (4).

(4) $$Ct=\frac{ktC^{2}s}{1+ktCs}$$

(5) $$\frac{t}{Ct}=\frac{1}{kC^{2}s}+\frac{t}{Cs}$$

### 3.10. Statistical Analysis

All experiments were performed three times to ensure the reliability of the results. GraphPad Prism 8.0.2 software was used to generate graphs of the experimental data.

## 4. Conclusions

This study provides a novel, green, and efficient method of extracting phenolic compounds from CLs using ultrasound-assisted technology combined with Pr-LA. Compared with traditional solvents, Pr-LA is the most advantageous extraction solvent for extracting phenolic compounds from CLs. SEM and FT-IR both confirmed that Pr-LA can enhance the degree of fragmentation of cell structures and improve the extraction yield of phenolic compounds. Molecular dynamics simulation results show that Pr-LA can improve the solubility of phenolic compounds and have stronger hydrogen bonds and van der Waals interactions with phenolic compounds, which also explains why the extraction efficiency of Pr-LA is higher than that of traditional solvents. The Box–Behnken design was used to optimize the extraction process of TPC from CLs using ultrasound-assisted combination with Pr-LA. Under the optimal conditions of a NADES water content of 41%, extraction time of 40 min, extraction temperature of 36 °C, and L/S ratio of 76 mL/g, the optimal extraction yield was predicted to be 24.93 mg GAE/g DW, and the actual extraction yield was 25.17 mg GAE/g DW, the experimental results are in good agreement with the predicted results. Finally, the kinetic analysis of the extraction process was performed, and the results show that the second-order kinetic model can be used to describe the extraction process of phenolic compounds from CLs, providing theoretical guidance for actual industrial production. In summary, ultrasound-assisted extraction combined with Pr-LA is a green, efficient, and economical method to extract phenolic compounds from CLs. However, this study needs to conduct further pilot scale-up experiments in the future and then apply the experiments to actual industrial-scale production. Of course, the safety of Pr-LA and Pr-LA CL extracts still need to be verified in the future so that it can be used more safely in food, medicine, cosmetics, and other fields.

## Figures and Tables

**Table 1:** Composition of natural deep eutectic solvents (NADESs).

DES No.	Abbreviation	Component 1	Component 2	Molar Ratio
1	ChCl-EG	Choline chloride	Ethylene glycol	1:2
2	ChCl-1,4-But	Choline chloride	1,4-Butanediol	1:2
3	ChCl-1,2-Pro	Choline chloride	1,2-Propanediol	1:2
4	ChCl-Gly	Choline chloride	Glycerol	1:2
5	ChCl-LA	Choline chloride	Lactic acid	1:2
6	ChCl-LevA	Choline chloride	Levulinic acid	1:2
7	ChCl-Urea	Choline chloride	Urea	1:2
8	Pr-LA	L-proline	Lactic acid	1:2
9	Pr-LevA	L-proline	Levulinic acid	1:2
10	Be-LA	Betaine	Lactic acid	1:2
11	Be-LevA	Betaine	Levulinic acid	1:2

**Table 2:** The results of the analysis of variance (ANOVA).

Source	Sum of Squares	df	Mean Square	F-Value	*p*-Value	
Model	177.56	14	12.68	24.11	<0.0001	significant
A	21.36	1	21.36	40.60	<0.0001	
B	3.71	1	3.71	7.05	0.0188	
C	4.07	1	4.07	7.74	0.0147	
D	30.51	1	30.51	57.99	<0.0001	
AB	5.69	1	5.69	10.81	0.0054	
AC	3.15	1	3.15	6.00	0.0281	
AD	2.86	1	2.86	5.43	0.0352	
BC	11.79	1	11.79	22.42	0.0003	
BD	0.9336	1	0.9336	1.77	0.2041	
CD	0.0151	1	0.0151	0.0288	0.8677	
A^2^	79.53	1	79.53	151.18	<0.0001	
B^2^	22.79	1	22.79	43.33	<0.0001	
C^2^	2.72	1	2.72	5.16	0.0394	
D^2^	15.77	1	15.77	29.97	<0.0001	
Residual	7.36	14	0.5260			
Lack of Fit	5.98	10	0.5979	1.73	0.3155	not significant
Pure Error	1.39	4	0.3464			
Cor Total	184.93	28				
R^2^	0.9602					
Adjusted R^2^	0.9204					
Predicted R^2^	0.8021					
C.V.%	3.43					

## Data Availability

The data presented in this study are available upon request from the corresponding author.

## Supplementary Materials

The following supporting information can be downloaded at: https://www.mdpi.com/article/10.3390/molecules29102385/s1, Figure S1: LC-MS total ion chromatogram of Pr-LA extract in the negative ion mode; Figure S2: Interactive effects of independent variables on TPC in extracted CLs; Table S1: 10 Phenolic compounds in Pr-LA extract identified by the LC-MS method; Table S2: Box–Behnken design and results for the UAE of TPC from CLs; Table S3: Kinetic parameters of the second order kinetic model for extraction.
