# Supplementary material for: Ultrasound-Assisted Extraction of Phenolic Compounds from Celtuce (Lactuca sativa var. augustana) Leaves Using Natural Deep Eutectic Solvents (NADES): Process Optimization and Extraction Mechanism Research
Source: Molecules. 2024 May 19;29(10):2385. doi: 10.3390/molecules29102385 (PMC11124495; doi:10.3390/molecules29102385)
Supplement: Supplementary file 1 [file molecules-29-02385-s001.zip › Supplementary Table S1.pdf]

Table S1 10 Phenolic compounds in Pr-LA extract identified by LC-MS method.

Peak no.	t_R(min)	Proposed compound	Molecular formula	$[M-H]^-$ (m/z)	MS/MS (m/z)	ID based on
1	5.28	Gallic acid	$C_7H_6O_5$	169.0131	169.0131	Standard
2	7.33	Chlorogenic acid	$C_{16}H_{18}O_9$	353.0867	191.0553,162.0233	Standard
3	8.38	Catechin	$C_{15}H_{14}O_6$	289.0706	109.0284, 245.0819	[43]
4	8.69	Caffeic acid	$C_9H_8O_4$	179.0339	135.0439	Standard
5	11.91	Rutin	$C_{27}H_{29}O_{16}$	608.1372	609.1491,300.0280,271.0254,609.1491	Standard
6	13.88	Isoquercitrin	$C_{21}H_{20}O_{12}$	462.0793	271.0252, 300.0280,301.0265	[45]
7	14.08	Ferulic acid	$C_{10}H_{10}O_4$	193.0495	149.0596, 178.0262	[47]
8	15.02	Luteolin	$C_{15}H_{10}O_6$	285.0765	133.0282, 151.0029	Standard
9	15.65	Naringenin	$C_{15}H_{12}O_5$	271.0618	119.0489,151.0024	[49]
10	15.83	Rhamnetin	$C_{16}H_{12}O_7$	315.0499	193.0458, 165.0369.	[50]
