# Supplementary material for: Ultrasound-Assisted Extraction of Phenolic Compounds from Celtuce (Lactuca sativa var. augustana) Leaves Using Natural Deep Eutectic Solvents (NADES): Process Optimization and Extraction Mechanism Research
Source: Molecules. 2024 May 19;29(10):2385. doi: 10.3390/molecules29102385 (PMC11124495; doi:10.3390/molecules29102385)
Supplement: Supplementary file 1 [file molecules-29-02385-s001.zip › Supplementary Table S2.pdf]

Table S2 Box-Behnken design and results for the UAE of TPC from CLs.

Run	Water content, A (%)	Extraction time, B (min)	Extraction temperature, C (℃)	L/S ratio, D(mL/g)	Extraction yield (mg GAE/g DW)
1	50	40	40	70	19.7555
2	50	40	30	80	18.2533
3	30	30	30	70	18.0255
4	40	30	40	70	23.7396
5	30	40	20	70	22.9465
6	40	50	30	60	19.9548
7	50	50	30	70	16.6747
8	40	40	40	60	19.6323
9	40	40	30	70	24.2236
10	40	30	20	70	18.7543
11	50	40	20	70	15.5864
12	40	50	40	70	20.5975
13	30	40	30	80	21.3048
14	40	40	30	70	23.4042
15	40	30	30	60	18.5729
16	40	50	20	70	23.1806
17	40	50	30	80	22.3168
18	40	40	20	60	22.0042
19	40	40	30	70	26.0502
20	40	40	40	80	24.0432
21	30	40	30	60	20.0451
22	50	30	30	70	16.9319
23	50	40	30	60	14.4123

24	40	40	30	70	24.4221
25	40	30	30	80	22.3673
26	40	40	20	80	22.669
27	30	50	30	70	19.5373
28	40	40	30	70	24.2236
29	30	40	40	70	18.5637
