# Supplementary material for: Ultrasound-Assisted Extraction of Phenolic Compounds from Celtuce (Lactuca sativa var. augustana) Leaves Using Natural Deep Eutectic Solvents (NADES): Process Optimization and Extraction Mechanism Research
Source: Molecules. 2024 May 19;29(10):2385. doi: 10.3390/molecules29102385 (PMC11124495; doi:10.3390/molecules29102385)
Supplement: Supplementary file 1 [file molecules-29-02385-s001.zip › Supplementary Table S3.pdf]

Table S3 Kinetic parameters of the second order kinetic model for extraction.

Process	Cs ($mg \cdot g^{-1}$)	k ($g \cdot min^{-1}\ mg^{-1}$)	R^2
Water	8.50991	0.02536	0.99022
Ethanol (50%)	15.6814	0.02697	0.99682
Pr-LA	24.3665	0.01048	0.99644
