# Supplementary figures and images for: Ultrasound-Assisted Extraction of Phenolic Compounds from Celtuce (Lactuca sativa var. augustana) Leaves Using Natural Deep Eutectic Solvents (NADES): Process Optimization and Extraction Mechanism Research
Source: Molecules. 2024 May 19;29(10):2385. doi: 10.3390/molecules29102385 (PMC11124495; doi:10.3390/molecules29102385)

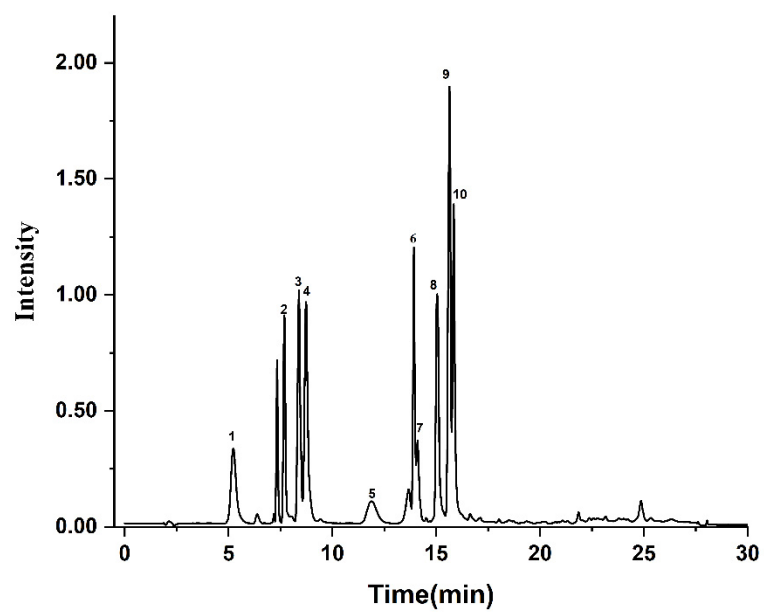

**Figure S1 LC-MS total ion chromatogram of Pr-LA extract in negative ion mode.**

Supplement: Supplementary file 1 [file molecules-29-02385-s001.zip › Supplementary Figure S1.pdf]

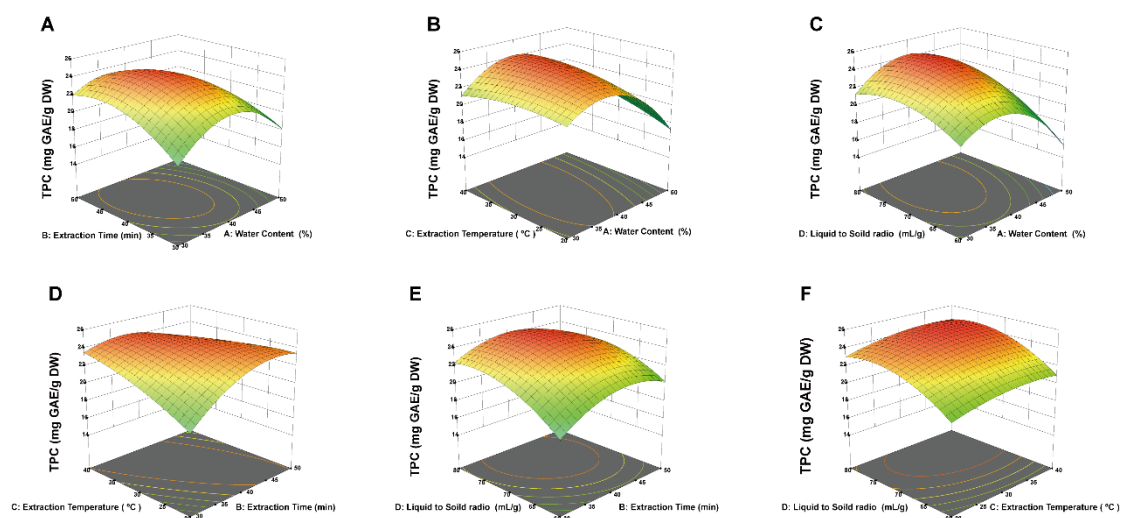

**Figure S2 Interactive effects of independent variables on TPC in extracted CLs.**

Supplement: Supplementary file 1 [file molecules-29-02385-s001.zip › Supplementary Figure S2.pdf]
